# Computational challenges in the analysis of ancient DNA

**DOI:** 10.1186/gb-2010-11-5-r47

**Published:** 2010-05-06

**Authors:** Kay Prüfer, Udo Stenzel, Michael Hofreiter, Svante Pääbo, Janet Kelso, Richard E Green

**Affiliations:** 1 Max-Planck Institute for Evolutionary Anthropology, Deutscher Platz 6, 04103 Leipzig, Germany; 2 Evolutionary Biology and Ecology, Department of Biology, University of York, York YO10 5YW, UK

## Abstract

High-throughput sequencing technologies have opened up a new avenue for studying extinct organisms. Here we identify and quantify biases introduced by particular characteristics of ancient DNA samples. These analyses demonstrate the importance of closely related genomic sequence for correctly identifying and classifying *bona fide *endogenous DNA fragments. We show that more accurate genome divergence estimates from ancient DNA sequence can be attained using at least two outgroup genomes and appropriate filtering.

## Abstract 2

A new method of next-generation sequencing analysis is presented which takes into account the biases characteristic of ancient, including Neandertal, DNA samples.

## Background

Most of our understanding of how extinct species are related to living species has come from morphological analysis of fossil remains. Recovery and analysis of DNA extracted from fossil remains, so called 'ancient DNA', provide a complementary avenue for understanding evolution. Analysis of ancient DNA has been used to resolve the genetic relationships between extinct and extant species [1-5], and to deduce extinct organisms' geographic ranges [6], and their phenotypic characteristics [7,8].

With the enormous throughput of next generation sequencers, it has become tractable to simply shotgun sequence DNA as it is recovered from fossil bones [9-13]. Despite the fact that most of the recovered DNA is from microbes that colonized the bone after death [4,14], the sheer volume of sequence generated means that the few percent that are typically from the species of interest still constitute a sequence dataset large enough for genome-scale analysis. Furthermore, because ancient DNA molecules are often fragmented to very short pieces [15], ancient DNA sequencing is not limited in practice by the short read length of current sequencers. The mean ancient DNA fragment length has varied between 60 and 150 bp in most recent large-scale sequencing studies [9-11,13,16-18], but can vary greatly from sample to sample.

Along with the obvious benefits of shotgun sequencing of ancient DNA, there are also new pitfalls. The presence of a large proportion of DNA from bacteria and other non-target species means that one must first identify the relevant DNA molecules from this complex background - a consideration not relevant to PCR-based methods. This is usually done by similarity searching using both the genome of a closely related species and large databases of microbial sequences. However, this search can fail to classify a molecule for one of several reasons. First, DNA sequences from ancient DNA often contain misincorporations stemming from base damage [12,19-21]. These errors could potentially result in spurious similarity, or more often, failure to detect similarity. Second, as noted above, ancient DNA fragments are generally quite short [11,15] and may not, therefore, have sufficient similarity to be correctly identified. Third, the databases of microbial sequences used to identify background sequences include only a small proportion of microbes found in nature [14]. Finally, the target genome used for detection of fragments of interest may not be sufficiently similar to that of the extinct organism to allow unambiguous detection of all relevant sequences. This last problem can be exacerbated by the heuristics used in fast database search programs, like BLAST [22].

The several recent analyses of ancient DNA shotgun data have largely deployed *ad hoc *methods to deal with these issues [9-11,13,17]. While necessity has required the use of fast local alignment programs such as BLAST [23], Mega BLAST [24] or BLASTZ [25] when handling such large datasets, the exact classification and filtering regimes have not been standardized or even comprehensively examined. In the most straight-forward classification scheme, reads that match a specific target genome with sufficient similarity are classified as endogenous (that is, from the target species) [11,13]. A simple extension of this method considers whether better alignments to other sequence databases exist, and use these to exclude potential microbial or other contaminants [9,10,17]. Divergence can then be calculated in a pairwise manner from the average similarity of all alignments for the sequences deemed to be endogenous [11,13,17]. Alternatively, in cases where an additional outgroup genome is available, such as the chimpanzee genome for the human/Neandertal comparison, a parsimony approach can be used to assign sequence differences to lineages. From such alignments a more reliable divergence estimate can be derived (later discussed in more detail) [9,10].

Here we identify and explore the biases introduced by the characteristics of ancient DNA when analyzing next-generation shotgun sequencing data. Since the primary goal of many projects is to resolve the genetic relationship between extinct and extant species, we focus our analysis on the classification of endogenous fragments (defined here to mean the DNA remaining from the bone's original owner and not from microbes or other external sources of DNA) and the calculation of pairwise nucleotide differences and divergence. We quantify the biases for these measures by using simulated as well as real Neandertal ancient DNA shotgun data. We find that a close genomic reference sequence is imperative when using standard alignment software. Our analysis leads us to identify a set of extinct species that may be considered tractable for informative ancient DNA shotgun sequencing.

## Results

To assess the biases introduced in the analyses of ancient DNA, we use a subset of the sequence data generated as part of the Neandertal genome project: 2.8 million reads from a 38,000-year-old Neandertal fossil bone [9,10,16] produced by shotgun 454 sequencing [26] on the GS FLX platform. Neandertal data are well suited for investigating the potential effects of having a progressively more distantly related comparison genome, since complete genome sequences are available from three great apes and several more distantly related primates. By using only the increasingly more distantly related genome sequences of human [27], chimpanzee [28], orangutan, rhesus macaque [29], mouse lemur, bushbaby and mouse [30], we gauge how many Neandertal sequences could be identified if each of these genomes was the only one that was available. We also investigated the accuracy of the observed number of pairwise nucleotide differences in each of these comparisons ([31].

Using a model of ancient DNA fragmentation and deamination [19], we also simulated datasets of 100,000 fragments with levels of difference corresponding to 1 to 6 million years of divergence from the human lineage. The simulation facilitates two types of analysis. First, since all fragments are simulated as endogenous hominin sequence, we can estimate how many endogenous fragments are lost during the various steps of alignment and filtering that precede further analyses. Second, with the actual amount of sequence divergence known from the simulation, we can directly compare our divergence estimates to discover and quantify biases. From these comparisons, we explore the effectiveness and accuracy of various filtering and alignment procedures to arrive at a reliable divergence estimate.

### Detection of endogenous fragments

The first step in the analysis of shotgun ancient DNA data is to identify the target-species (endogenous) fragments. The primary goal of this step is to reliably identify as many endogenous fragments as possible. Ideally, this identification would not introduce major biases that would skew subsequent analyses.

Theoretically, there are two ways to detect endogenous fragments if only microbial contamination is present. First, microbial sequences could be initially identified and then subtracted. Any non-microbial sequences would therefore be sequences from the target species. Alternatively, endogenous fragments could be detected by similarity to a related genomic sequence. While the first method is preferable insofar as it would allow the detection of novel sequences and highly diverged regions between the target species and any comparison genome, recent studies indicate that currently available microbial sequence data are too incomplete to detect the full diversity naturally occurring in microbial communities [14,32]. Therefore, the only currently practical way to identify target-species DNA fragments is by similarity between these and the sequence of a closely related species. For example, Neandertal sequences are identified based on their similarity to the human or chimpanzee genomes and mammoth sequences are identified based on the similarity to the elephant genome [9-11,13,17]. The specificity of this approach can be increased by further requiring that similarity to a closely related genome is higher than similarity to any known microbial sequence [9,17].

Because of the generally low percentage of endogenous fragments, especially from less well preserved, non-permafrost-derived specimens such as Neandertal bones, extensive sequencing is necessary to recover enough fragments for subsequent analyses. This, in turn, requires substantial computing power to carry out similarity searching against multiple genome databases. Several widely used local alignment programs provide fast comparison of sequences to large databases by requiring a short exact-matching sequence (seed) to start the alignment [22,33]. This heuristic speeds the search-time since computationally expensive alignment is restricted to sequences that share at least a short seed. However, the exact-match seeds that trigger alignment become rarer at greater evolutionary distances [34], precluding identification of some similarities. This erosion of sensitivity is exacerbated in ancient DNA shotgun data since, in addition to the divergence to the genome used for comparison, chemical damage to the molecules results in shorter read lengths and erroneous bases. For our analysis, we seek to minimize this effect by setting the seed size as short as computationally feasible. We use a contiguous seed size of 16 for Mega BLAST [24].

Using our Neandertal dataset we measured the number of fragments identified as Neandertal by using increasingly distant genomes for similarity searching. These genome sequences span a range from less than 1 million years (between Neandertal and human) [9,10] up to 87 million years of divergence (between mouse and human) [35]. Mouse-human genome divergence has been estimated to be, on average, 0.5 substitutions per site [30]. This constitutes the most diverged genome comparison in our test. Using each of these genomes as the search target, we asked how many sequences are identifiable as Neandertal. In this way, we can directly assess the cost of increasingly distantly related comparison genomes in terms of lost sensitivity.

**Figure 1:**
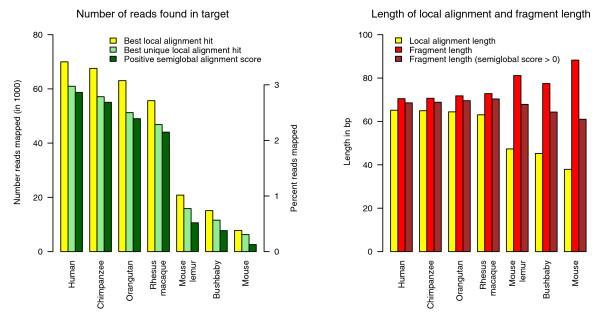
**Number of aligned ancient DNA fragments and average sequence length**. Properties of Mega BLAST alignments of ancient DNA sequences from a Neandertal fossil to genome sequences of increasing divergence. Left panel: number of reads with a best hit to the genome sequence and not to the GenBank nonredundant and environmental databases (yellow). Subset of reads with one unique best hit to the reference genome (light green). Subset of reads with one unique best hit to the reference genome that can be fully aligned with a positive alignment score (dark green). Right panel: Average length of best local alignments (yellow), average length of fragments with a unique best local alignment (red), average length of fragments with a positive score when fully aligned to reference genome (brown).

When we used the human genome as the reference sequence, we estimated a total of 69,959 reads (or 3.4%) to be of Neandertal origin. A further 13.6% of all reads could be classified based on similarity to a non-human sequence in GenBank, including microbial data in the nonredundant and environmental databases. The majority, 83%, had no significant similarity (e-value <0.001) to any database sequence. This same procedure was then carried out substituting the chimpanzee, orang-utan, rhesus macaque, bushbaby, mouse lemur and mouse genomic sequences, respectively, for the human genome sequence. As expected, both the number of fragments identified and their local alignment length decrease (Figure 1a, b) as more distant genomes are used for searching and alignment. Both observations are attributable to the alignment algorithm used. First, the shorter local alignments are caused by the extension algorithm of the local alignment program, which extends the alignment only as long as the score does not drop by a certain value below the previous maximal score by aligning further bases [22,24]. The extension of the alignment will therefore stop earlier if the target genome is more distantly related, thus leading to shorter local alignments. Second, a fragment will remain undetected if no seed match is found to start the alignment. Similarly, reads may fail to produce an alignment with a score high enough to trust.

Although the average alignment length decreases with increased evolutionary distance, the length of the fragments on which these alignments are found increases (Figure 1b). However, this seemingly paradoxical result can be explained in the following way. The chance of finding a seed-match and of producing a local alignment of significant similarity rises with the length of the fragment. Longer fragments, then, are more likely to have a seed sequence and therefore to be detected as Neandertal. In summary, local alignment programs such as Mega BLAST or BLAST produce alignments that cannot be taken at face value as a description of the percentage or lengths of endogenous ancient DNA sequences in a sample, especially when the alignments are against a distantly related genome sequence.

To characterize identifiable ancient Neandertal sequence fragments more fully, we explored the effect of simply extending these local alignments to include the entire sequence. Because of the library construction method, we know that recovered sequences represent a single contiguous segment of DNA from the DNA extract, that is, they are not chimeric. These sequences should thus be aligned globally with respect to the ancient sequence, not locally as is done using Mega BLAST. We therefore implemented a semi-global alignment algorithm that is global with respect to the fragment, local with respect to the genomic sequence, and is seeded by the initial local alignment. The scoring scheme for this alignment uses affine gap costs [36]. Only sequences with one uniquely best hit to the target genome were semi-globally aligned, since the right location for multiple equally good hits is unknown. This introduces a possible complication if the local alignment represents spurious similarity embedded within otherwise unrelated sequence or if an indel or other rearrangement has occurred in the evolutionary time separating Neandertals and the compared species. To avoid analyzing such sequences, we required that the overall semi-global alignment score remains positive, that is, that the sequence left unaligned by the local procedure was not so dissimilar as to render the semiglobal alignment more likely to occur by chance than by true evolutionary relatedness. Using this alignment procedure, the fraction of positively scoring alignments decreased with the degree of divergence from the reference genome (Figure 1a). However, the fragment length of positively scoring alignments remains more constant at increasing evolutionary distance (Figure 1b). Therefore, this alignment procedure gives a more accurate depiction of the length of endogenous ancient fragments than simple local alignment length in cases where the closest comparison genome is evolutionarily distant.

### Pairwise differences

Once endogenous reads are identified, their alignments can be examined to calculate the average number of differences per site. However, there are several complications for this analysis that are specific to ancient DNA. First, unrelated microbial sequence may be falsely classified as endogenous. Second, truly endogenous reads that are highly diverged may not be identified as such. Third, endogenous reads may be correctly identified, but incorrectly aligned, for example by being placed at a paralogous region. Finally, post mortem DNA damage manifests in miscoding lesions. Each of these complications can bias the number of pairwise differences: failure to identify highly divergent reads results in pairwise differences being biased downwards while the other factors will result in an upward bias. Given theses sources of error, we investigated the reliability of observed pairwise nucleotide differences with respect to increasing evolutionary distance.

From the alignments described in the previous section, we calculated the differences between Neandertal sequences and the genomic sequence of species of increasing evolutionary distance. For comparison, we also calculated the pairwise nucleotide differences between humans and several other species spanning an identical range of divergence using the data from randomly picked genomic regions provided by the ENCODE project [37]. These much larger regions were previously sequenced and aligned using the alignment program MAVID [38]. This dataset has the advantage that each region contains sequences with one-to-one orthology between humans and the other aligned species and is in this respect similar to our pairwise sequence alignments. However, difference estimates given by the MAVID alignment of these randomly picked ENCODE regions can potentially contain a technical bias [39] and are not to be taken as absolute truth. For our purposes, they are simply a convenient way of measuring the general trend of increasing pairwise sequence differences between evolutionarily more distant species. For this analysis, we do not use a correction for multiple substitutions. Since our goal is to quantify the effects of various sources of error, the interaction between these errors and more refined pairwise divergence measures would make the results harder to interpret.

**Figure 2:**
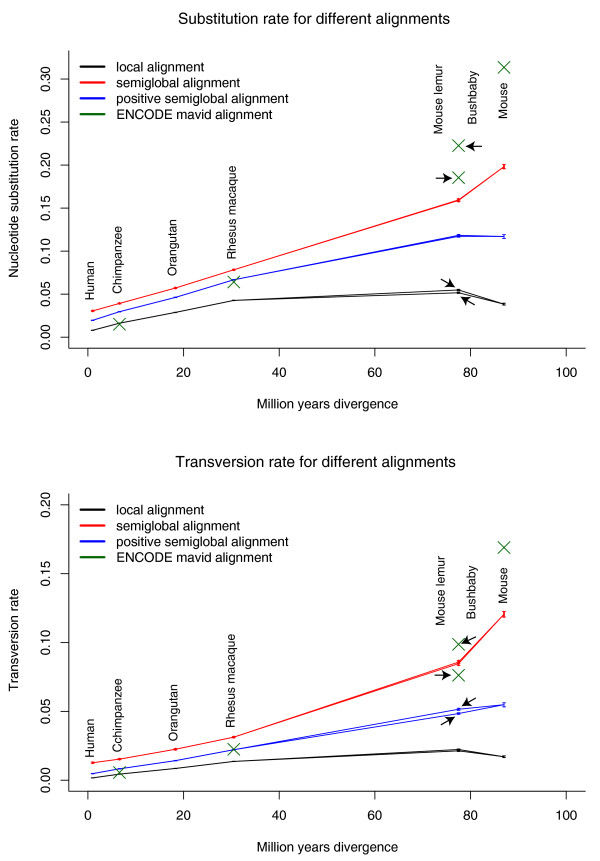
**Differences per site in alignments of ancient DNA fragments**. All nucleotide differences (top) and transversion differences (bottom) in different alignments to reference genomes of increasing divergence. Each read is required to have one uniquely best Mega BLAST alignment to the reference genome (estimate shown as the black line). The semiglobal alignment forces the full sequence to align to the genomic region identified by the local alignment (estimate shown as red line). These full alignments are further filtered for having a positive alignment score (blue line). The green crosses show the differences between human and the reference species in the ENCODE multiple sequence alignments. The divergence times on the x-axis are from [52] and [35], except for human for which we choose an arbitrary divergence time of 1 million years to Neandertal.

For each comparison genome, we found that the observed number of differences per site in the local alignments was lower than the value measured from the ENCODE alignments. Notably, the observed pairwise differences even decreased at the most extreme evolutionary distance, that is, to mouse (Figure 2). As discussed previously, since local alignments are not extended into regions of dissimilarity that decrease the overall alignment score, this result can easily be explained. Dissimilar regions are simply left unaligned. Using the full semi-global alignments to measure pairwise differences per site yields values that are more consistent with the ENCODE alignments at increasing evolutionary distance. We also explored the effect of filtering semi-global alignments for positive score. Unfiltered semi-global alignments to mouse show a substantially lower number of differences compared to the differences calculated from ENCODE regions. The low number of differences is primarily caused by the first step of the analysis: the identification of Neandertal sequences. The Mega BLAST method, used in this step, is intended for the comparison of longer, closely related sequences [24] and will inevitably fail to detect some of the more divergent reads. This bias against identifying and aligning more divergent reads, in turn, leads to the low number of differences. We observe the opposite effect for alignments to chimpanzee where all alignment procedures showed a higher number of differences than reported for the ENCODE regions. Part of this effect is attributable to ancient DNA damage. Overrepresentation of C->T and G->A transitions in ancient DNA sequencing data was previously described as the main result of miscoding lesions [12,19-21]. These changes cluster primarily at the 3' and 5' end of the molecules, probably due to single-stranded overhangs that are more susceptible to deamination at the end of the sequenced molecules [19]. These properties will affect semi-global alignments more than local alignments, since the former include the full ancient DNA sequence, including the ends where these misincorporations are abundant. We therefore restricted the analysis to transversions and recalculated the number of differences for all reference species and ENCODE regions (Figure 2b). The number of transversion differences for semi-global alignments with a positive score follows the general trend of transversion differences of ENCODE region alignments for rhesus macaque and chimpanzee. The value for rhesus macaque is in closest agreement with the expectation from the ENCODE alignments. The number of transversion differences to chimpanzee is about 48% higher for the semi-global filtered alignments and 21% lower for local alignments than the number of transversion differences in randomly picked ENCODE region alignments. This demonstrates the difficulties with direct pairwise comparisons, and highlights the need for using an outgroup sequence to the ancient genome and the closest related genome for measuring divergence as discussed in the following section.

### Divergence triangulation

**Figure 3:**
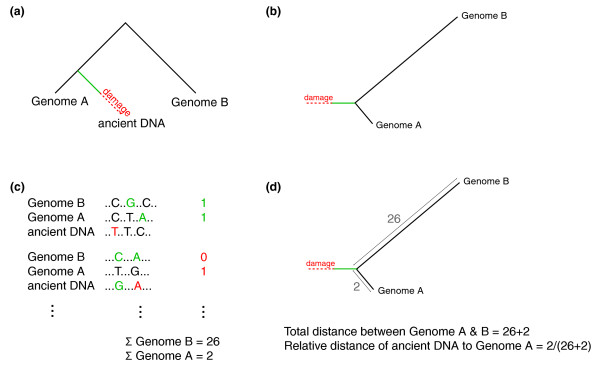
**Schematic description of divergence triangulation**. **(a) **A phylogenetic tree depicting the necessary topology for the application of the divergence triangulation method. **(b) **The ancient DNA sequences are used like an outgroup to the two genomic sequences in an unrooted tree. **(c) **Alignments between genomic sequences and ancient DNA fragments are used to assign changes to the lineages (numbers on the right-hand side). In this process, coinciding changes often caused by ancient DNA damage (shown in red in the alignments) can lead to misassignments of differences (in red in the summary of tables) **(d) **The assigned differences can be used to calculate a divergence relative to the divergence between the two genome sequences.

In cases where the genome sequences of two closely related species are available and one of them is known to be more closely related to the ancient species than the other, additional comparisons are possible that can mitigate the biases in estimates of divergence inherent to ancient DNA. Neandertals are one species where two close genome sequences are available: human and chimpanzee. In a three-way comparison, substitutions can be partitioned onto the respective lineage on which they occurred. Those that are specific to Neandertal, which include ancient DNA associated nucleotide misincorporations and other sequencing errors, can be ignored (Figure 3). This method conveniently provides an estimate of the number of changes along the lineages to both human and chimpanzee genomes in an unrooted tree, and largely circumvents the problem of nucleotide misincorporations as these are isolated on the Neandertal lineage. That is, at these positions, the Neandertal base will match neither human nor chimpanzee (except in the rare instance of a parallel substitution in either human or chimpanzee that mirrors the nucleotide misincorporation in the Neandertal sequence). Assuming a molecular clock, the ratio of the number of changes specific to the human lineage to those specific to the chimpanzee lineage gives an estimate of the Neandertal-human divergence. With prior knowledge of the divergence time between the human and chimpanzee genomes, a divergence time can in turn be assigned to this branch point. This method has been previously used to estimate the Neandertal-human divergence time based on alignments to human and chimpanzee sequences [9,10].

Compared to divergence estimates based on the observed differences in a pairwise alignment, this method of divergence triangulation has a number of advantages. As described above, misread bases in ancient DNA will lead to an overestimate of divergence in a pairwise comparison. However, since the ancient DNA sequences are used to assign changes to lineages, an error in this sequence will only bias the divergence estimate if it occurs at a site with an independent change in either of the two genomic sequences. Also, while a bias against highly diverged sequences will lead to an underestimate of divergence in a pairwise comparison, the divergence estimate in the triangulation method remains stable as long as the bias affects both genomes equally.

We used the simulated datasets to test the stability of the triangulation method and to devise further filtering methods to increase its accuracy. The simulated fragments were generated to match the observed length distribution of ancient Neandertal fragments. Each simulation set also had a fixed average divergence built-in using data from the available human-chimpanzee whole genome alignments [40]. To complete the simulation, we added lineage-specific and ancient DNA-associated substitutions to model what is observed in actual ancient DNA (see Materials and methods). We then compared various approaches of the triangulation method to estimate human/Neandertal divergence and compare this estimate to the known divergence engineered into the simulated Neandertal sequences.

We aligned the simulated sequences to the human and chimpanzee genomes and the GenBank non-redundant and environmental databases using Mega BLAST. For our purpose, alignments to both the human and chimpanzee genomes are required for the subsequent steps of analysis and filtering. Around 99% of the reads consistently passed this criterion for all simulated datasets. The vast majority of the remaining reads had no significant local alignment to any of the databases searched, or failed to align to either the chimpanzee or human genome. Only a small percentage (less than 0.1% for all datasets, in agreement with our e-value cutoff) was misclassified as a result of having a best hit to a non-primate sequence.

When short reads are aligned to more distantly related genomes, these reads fail to be correctly identified as Neandertal more often than longer reads [41]. For the triangulation method, this effect can cause a bias in the divergence estimate when it is primarily highly diverged reads that cannot be mapped. This bias further depends on the method used to construct the multiple sequence alignment. When the multiple sequence alignment is constructed by aligning the ancient sequence reads to the genome of species A to identify endogenous reads and then species B is added to the alignment using a whole genome alignment between the genome sequences of A and B, the selective bias against highly diverged reads will lead to an apparent closer relationship between the extinct species sequence and the genome used for identification (species A). For our simulated datasets of 1 to 6 million years, the number of unidentified reads after alignment to the human genome is generally small and constitutes the largest part in the size fraction below 35 bp (Figure S1 in Additional file 1).

A multiple sequence alignment can also require independent alignments to the genome sequences of both species A and species B. In this case, the bias can only influence the divergence estimate if it affects one of the two alignments more strongly than the other. This is the case if there are more pairwise differences to one of the genome sequences than to the other. Our dataset simulating one million years of human-Neandertal divergence shows such a difference and we used it to test for this bias. A total of 1,130 (1.1%) fragments failed to align to either extant species' genome in this dataset. Of these, 988 simulated sequences failed to align only to chimpanzee but had a significant alignment to human, while 47 fragments had no significant alignment to human but aligned to chimpanzee. When we consider all fragments that fail to align, we observe that these fragments show a simulated divergence of 0.66 million years (confidence interval 0.54 to 0.79) to human. Therefore, the local alignment procedure causes a biased subset with high divergence to chimpanzee to be lost for further analysis. However, since only a small fraction of reads cannot be used, the effect on the divergence estimate from the remaining data is negligible; the divergence estimate for reads with alignments to both human and chimpanzee differs by less than 1% from the simulated divergence. The average size of fragments without alignment to human and chimpanzee genomes, 54 bp, was slighter shorter than the average size of 63 bp. This suggests that a size cutoff could be used to alleviate this bias.

Apart from these two effects, a size cutoff is often necessary to identify and exclude other mammalian contamination from ancient DNA analyses. In a test with mammoth DNA we observed that reads with a length of less than 30 bp often align best to a wide range of mammalian species, while longer sequences are almost exclusively identified as mammoth (data not shown). This indicates that reads of this size are too short to identify the originating species reliably. For this study, we evaluate the influence of a size cutoff of 35 bp.

Since the simulated fragments are used to partition human-chimpanzee differences, it is crucial to ensure that the aligned human and chimpanzee sequence is orthologous [41]. We used the whole genome alignments between the human and chimpanzee genome to map each uniquely best local alignment location with respect to the other genome (see Materials and methods for further details). Only hits that had an overlap between original and mapped location in both directions were kept for further analysis. About 88% of reads in each dataset passed this filter. Using the original genome location for each simulated fragment, we tested how many of the remaining fragments were not aligned to the orthologous position. Between 0.2 and 0.3% of the reads in the simulated dataset were misaligned after filtering. Since the reads align to a non-orthologous location, it is likely that a nearly equal second best alignment exists to the correct location or other similar regions. We find that over 95% of the reads aligning to a non-orthologous position produce two or more alignments to the human genome whose bitscores differ by less than 6 points (Figure S3 in Additional file 1). Therefore, requiring a minimum distance in bitscore between the best and second best hit is very effective in removing most of the remaining reads that would otherwise produce non-orthologous alignments.

**Figure 4:**
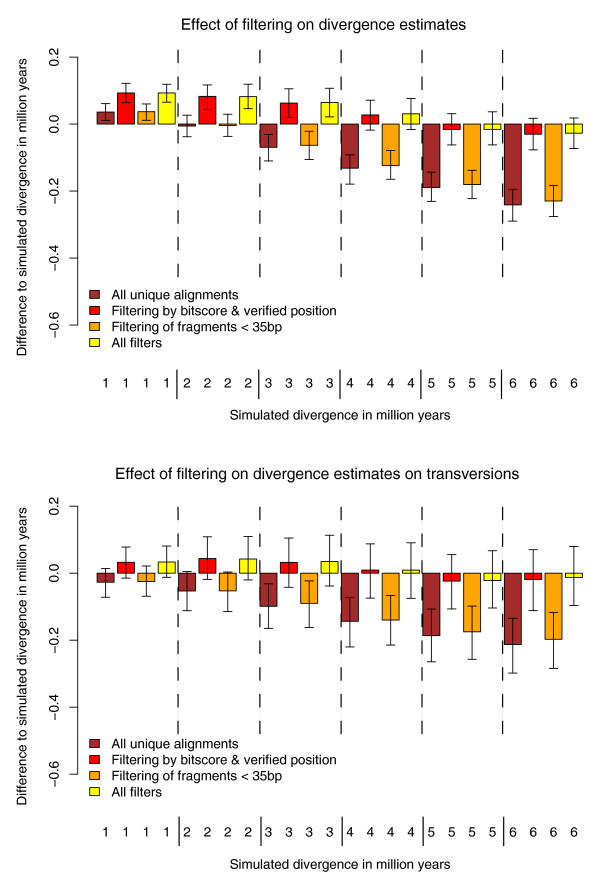
**Divergence estimates by triangulation on simulated datasets**. **(a) **3DP divergence estimates in comparison to the expected values. Four bars are drawn for different filters: raw estimate without filtering on all unique alignments (brown); filtered alignments with verified human and chimpanzee genomic location using a whole genome alignment and a distance of at least 6 points between best and second best local alignments' bitscores (red); alignments of fragments with a size >35 bp (orange); and all filters applied (yellow). **(b) **Estimates are derived solely from transversion differences, otherwise identical to (a).

With these observations in mind, we imposed various filters on each of the simulated datasets after aligning the human, chimpanzee and simulated Neandertal sequences using a full three-dimensional dynamic programming algorithm (3DP) to avoid bias introduced by progressive multi-sequence alignment. We then measured the deviation from the expected divergence given by the simulation parameters (Figure 4a). Unfiltered alignments result in an overestimate for lower simulated divergence and an underestimate for higher simulated divergence. Part of this effect can be explained by the different alignment procedures used to compose the multiple sequence alignments: while a unique local alignment to human is required, the chimpanzee sequence is added from a whole genome alignment. We tested the effect of our length filter excluding fragments below 35 bp. This filter gives slightly higher divergence estimates, with the most notable effect seen at higher simulated divergence times. Next, we tested the effect of filtering non-orthologous alignments using the unambiguous orthology filter and the bitscore filter. After applying these filtering procedures all divergence estimates increased. This led to an overestimate of divergence for small simulated divergence, while higher simulated divergence of 4 to 6 million years is in agreement with the simulated value. The combination of all filtering showed a similar deviation from the divergence modeled into these sequences.

The overestimated divergence for simulated data with a high difference in lineage length could be due to independent but identical substitutions in the simulated data and in one of the outgroup sequences, leading to misassignment of changes. Ancient DNA damage manifests as transitional differences in the ancient DNA sequence (C to T and G to A differences) and transitions are also observed as a frequent difference between human and chimpanzee. Therefore, this artifact is likely to occur by chance. If the branch point of the ancient sequence is not located centrally between the two comparison genome sequences, the genome with a higher true distance will have a greater chance of showing an independent change. This leads to an overestimate of the divergence to the more closely related genome. Since coinciding ancient DNA damage and independent chimpanzee changes are likely to occur more often for faster-evolving transitions, we repeated the calculation based on transversion differences. The 3DP alignments did not differ significantly from the expectation for divergence estimates based on transversions if all filtering procedures are applied (Figure 4b). Therefore, under the conditions of our simulation, a stable divergence estimate can be reached when applying appropriate filtering criteria to minimize the effect of biases in the alignments, misalignments to paralogous positions and coinciding independent changes.

### Evaluation of potential sequencing targets

Based on our results, we analyzed the feasibility of the whole genome shotgun approach on other extinct species. For this purpose, several criteria have to be taken into consideration. The first step, of course, is locating a sample containing endogenous DNA. Results from decades-long explorations of different fossils indicate that the presence of endogenous DNA depends on two main factors: age and preservation conditions. The oldest ancient DNA sequences obtained to date come from the silty section of an ice core from Greenland [42] and date to approximately 500,000 years. However, in warmer environments, DNA may degrade much more rapidly [43]. Due to these limitations, several potentially interesting sequencing targets are likely to be currently out of reach for ancient DNA research. These include the *Homo floresiensis *fossils that were found in a warm environment, likely precluding the preservation of endogenous DNA. Other archaic hominins such as *Australopithecus *whose extinction predates the oldest fossils that have yielded endogenous DNA are also likely intractable for ancient DNA work. On the other hand, endogenous DNA has been recovered from several younger or better preserved fossils from a wide range of species, such as cave bears, mammoth, mastodons or saber tooth cats.

When a well preserved fossil is identified and sequenced, a related genome sequence is needed to detect endogenous fragments and exclude contaminating sequences. As we have shown in our analysis, the number of fragments that can be identified as endogenous depends on how closely related this comparison genome sequence is. Apart from recovering more sequences for the analysis, a more closely related genome sequence also gives a more complete picture of the ancient genome by avoiding a bias against highly diverged regions. Correspondingly, the absence of a close living relative limits the value of a genome project of an extinct species as any sequence comparison will be limited to genomic regions that share sufficient conservation to reliably detect ancient DNA sequences. An example of such a species is the saber tooth cat. Although potentially interesting for its unique morphological characteristics, this species is relatively isolated in the phylogenetic tree (Figure S4 in Additional file 1). For this reason a genome project for the extinct saber tooth cat may be of limited value. However, closely related genomes are available for several other extinct species. The currently ongoing Neandertal Genome Project uses the human and chimpanzee genome sequences to identify endogenous Neandertal fragments and the recently published sequences from a mammoth were analyzed using the draft African elephant genome sequence. We have listed several other extinct species whose genome sequences would be biologically interesting, together with the closest living relative in Table 1.

**Table 1:** Evaluation of potential ancient DNA shotgun sequencing targets

Species	DNA preservation	Biological relevance	Closely related genome available
Neandertal	Yes, reasonable	Recent human evolution	Human, chimpanzee
Mammoth	Yes, very good. Draft genome published in 2008 [13]	Limited; possibly adaptation to arctic environments	Elephant
Mastodon	Yes, good	Limited; in combination with mammoth parallel adaptation to arctic environments	No close living relatives
Dwarf elephant	Maybe possible; young enough, but poor preservation conditions	Rapid decrease in body size due to island adaptation	Elephant
Cave bear	Yes, good	Limited; probably interesting in combination with genomes from modern bear species; long hibernation without muscle atrophy may be medically interesting	Bear (not sequenced)
Ground sloth	Yes, reasonable	Size difference to modern species; parallel evolution in different lineages	Tree sloth (sequencing in progress)
Saber tooth cat	Probably possible	Limited; unique morphological adaptations	No close living relatives
Aurochs (*Bos primigenius*)	Marginal; young enough, but poor preservation conditions in region of domestication	Understanding of domestication process	Cattle [53]
*Homo floresiensis*	No, young enough, but too poor preservation conditions	Relationship to modern humans; recent human evolution; island adaptation in a hominid	Human, chimpanzee
Australopithecus	No, too old	Human evolution: potentially medical insights	Human, chimpanzee
Dinosaurs	No, far too old	Unique evolutionary lineage	No close living relatives

## Discussion

Because of the generally low amount of endogenous DNA, ancient DNA shotgun sequencing projects will continue to depend heavily on how well endogenous reads can be identified, and thus on the availability of a closely related genome sequence. With the data and parameters used in our study, we see that only a small subset of primarily long reads is identified as endogenous when highly diverged comparison genome sequences are used. This problem is further exacerbated when the full ancient DNA sequence is aligned to identify and remove likely false positive hits. Using distant comparison genomes with many genome rearrangements or draft genome assemblies of lower coverage, when this is all that is available, will naturally lead to a further decrease in the number of reads that pass this filtering.

We also show that the measurement of pairwise differences per site is influenced by several factors. In particular, the heuristic used in local alignments can cause a bias towards an underestimate of differences and the consequent failure to discover interesting fast-evolving regions. This bias dominates when highly diverged genomes are used for comparison, which emphasizes the importance of having a closely related genome sequence for the detection of endogenous reads. In some cases, this bias can be alleviated by restricting the analysis to longer fragments [34]. On the other hand, an overestimate of differences can be caused by ancient DNA misincorporations, misassignment of endogenous reads to paralogous positions, and false positive alignments of microbial reads. A number of steps can be taken to minimize the effect of these factors. In our analysis we excluded ancient DNA misincorporations, which usually lead to transitions, by simply calculating only the number of transversions per site. Furthermore, as the fraction of endogenous reads is usually quite low and some amount of microbial sequences will be falsely assigned as endogenous, a close genome sequence is crucial as it allows identification of a larger fraction of the truly endogenous sequences. The same effect could, in principle, be achieved by using a sample with a high percentage of endogenous reads, as in the mammoth genome project [13]. However, it is frequently the case that no samples with a high percentage of endogenous DNA are available for an extinct species.

When genome sequences of two comparison species are available such that one represents an outgroup, the ancient DNA sequence can be used to assign sequence changes to specific lineages of both comparison species. Since our analysis of this methodology was conducted on a simulated dataset containing only endogenous reads, we cannot infer how much any analysis based on this method would be influenced by false positive alignments of microbial reads. However, we were able to show that filtering based on the second best alignments, and verification of the alignment positions through a whole genome alignment effectively removes reads aligning to non-orthologous sequence from further analysis. Furthermore, when excluding damage-associated changes and using a full three-way alignment procedure, the divergence estimates are reliable. For the parameters used for damage, fragment length and divergence times of 1 to 6 million years, the triangulation method can therefore be used to calculate divergence as long as no substantial bias is introduced by false positive alignments involving microbial sequences.

## Conclusions

The rapid pace of advancement in high-throughput sequencing, coupled with advances in ancient DNA extraction and library generation [44,45] have naturally spurred the field to dream about what is possible. While genome-scale ancient DNA data hold the promise of directly addressing fundamental questions about how extinct species evolved and adapted to their environments, there are very real obstacles to be overcome. Some of these obstacles, such as finding biological remains with intact DNA, are well known and largely a matter of chance. Other obstacles, such as the lack of a closely related, high-quality comparison genome sequence are surprisingly important but increasingly surmountable [46].

## Materials and methods

### Data

The simulated datasets are available in fasta format in Additional file 2 or can be downloaded from [47]. The Neandertal sequencing data used in our analyses have been deposited to the Sequence Read Archive under the accession ERP000126 [48].

### Initial processing of sequencing data (adapter trimming, clustering)

We filter the metagenomic 454 reads on the first four bases that encode for a Neandertal specific key sequence to filter for potential cross-contamination by reads from other 454 libraries. Our reads are not adapter-trimmed, to allow us to distinguish between quality trimmed sequence and fragments that are shorter than the read-length of the 454 FLX sequencer. We use an in-house developed program to remove the adapter sequence prior to alignment. Adapters were identified in flow space by comparing individual flow values starting at each possible trim point to those of the known adapter sequence. Equally strong flows score positively, differences in magnitude are penalized. The total score is normalized for the length of the overlapping region and the 5'-most trim point that scores positively is used to cut away the adapter.

As previously described, emulsion PCR can produce a substantial number of clusters of identical fragments if a low concentration of DNA is used [9]. We identify these emulsion PCR duplicates using the following algorithm: reads are sorted into buckets according to the first six positive flow values. A new cluster containing two reads from a bucket is formed if these reads have at least 89% sequence similarity over the full length of the shorter read including the 454 adapter sequence. A read is added to an existing cluster if the same condition is met by any one of the sequences in the cluster (single-linkage clustering). The algorithm identified 736,426 of a total of 2,796,944 reads, or 26%, to be duplicates of other sequences.

### Classification of reads through best local alignment

All reads (including all potential emulsion PCR duplicates) are aligned with Mega BLAST version 2.2.14 to the human (hg18), chimpanzee (panTro2), orang-utan (pongo 2.0.2), rhesus macaque (rheMac2), mouse lemur (micMur1), bushbaby (otoGar1) and mouse (musMus8) genomes and the GenBank non-redundant (nt, snapshot 2006-06-16) and environmental databases (env, same date). The used Mega BLAST parameters are: -b 10 -v 10 -U F -I T -e 0.001 -F F -W 16 -M 15000. The Mega BLAST output is parsed using a modified version of the libzerg library [49] and a table containing the best ten alignments between any pair of query and target fasta record is produced for each target database. An additional entry to the table with hits to the non-redundant GenBank database is added containing the taxonomic identifier (GenBank Taxonomy DB) for the target sequence.

For each target genome a list of best hits is generated by comparing the GenBank Database tables and the target genome database, by keeping the hit with the best bitscore. We exclude all primate GenBank non-redundant database hits (taxonomy ID 9443) when comparing to any of the target primate genomes and all hits to species in the super-order Euarchontoglires (taxonomic ID 314146) when comparing to the mouse genome to get exclusively hits to the target genome for reads that are classifiable as target. If multiple equally good hits to the target databases exist, the hit is flagged as non-unique.

The resulting best hit table is then further filtered to remove emulsion PCR duplicates. We keep the best scoring hit of all hits produced by reads of the same cluster.

### Semiglobal alignment and assessment of sequence divergence

We implemented an alignment algorithm that is global with respect to the query sequence, but local with respect to the database, following the method of [36]. To make full dynamic programming feasible, only a small part of the database around the known best local alignment is used as reference sequence.

### Simulating ancient DNA fragments

Fragments are picked randomly from the autosomal chromosomes of the human genome (UCSC Genome Browser release hg18). The fragments' lengths are sampled from the observed size distribution in a 454 Neandertal run after classification using the human genome sequence. The fragments are further filtered using the human-chimp and chimp-human whole genome alignments (between versions hg18 and panTro2, downloaded from UCSC Genome Browser) to ensure that an unambiguous alignment between the human and chimpanzee genomes exists. For this purpose we map the read coordinates to the chimp genome and back to the human genome using liftover [50]. Only fragments that are accurately mapped back to their original human coordinates are retained. A total of 100,786 simulated reads pass this filter and are used in the subsequent steps to simulate different divergence times.

For each alignment between human and chimpanzee we simulate Neandertal reads in four steps. In step 1, we start with the human sequence in each pairwise human-chimpanzee alignment. Given a divergence of 6.5 million years between the human and the chimpanzee sequence (and thus a total distance of 13 million years), we substitute the nucleotide in the human sequence by the chimpanzee variant with a probability of X/13, where X denotes the desired simulated Neandertal divergence. In step 2, random substitutions are added to this sequence. A nucleotide substitution matrix is calculated from the original 100,786 pairwise alignments between human and chimpanzee. With a probability of X/13 × r, with r being the average nucleotide substitution rate between human and chimpanzee, a nucleotide is mutated and the new nucleotide is picked according to the nucleotide substitution matrix. With the same procedure as used in step 1, gaps and insertions present in the chimpanzee sequence in the alignment are introduced into the simulated Neandertal sequence in step 3. In step 4, ancient DNA miscoding lesions are added to the sequence according to the model of ancient DNA damage by [19], using the following parameters: length of overhang according to a geometric distribution with parameter 0.3, a nick probability of 0.8, single-stranded DNA deamination rate of 0.845 and a double-stranded DNA deamination rate of 0.015.

We use this procedure to simulate a divergence of 1, 2, 3, 4, 5 and 6 million years assuming a divergence time of 6.5 million years between chimpanzee and human autosomal genome sequences.

### Alignments for the analysis using two close genomes

The simulated fragments produced by this method are subsequently processed using our standard pipeline for Neandertal reads. The reads are first aligned (with Mega BLAST) to the human, chimpanzee, rhesus and mouse genomes and the GenBank nonredundant and environmental databases. Reads are classified as Neandertal if the best local alignment (according to bitscore) is to a primate genome sequence. Reads with a unique best hit to the human genome are aligned semiglobally to include the full sequence. Next, the coordinates of this alignment on the human genome are used to extract chimpanzee sequence from human-chimpanzee whole genome alignment. Human, chimpanzee and simulated sequence are then aligned as described in [51]. The 3DP matrix is not filled completely, but instead traversed once using Dijkstra's Algorithm, giving the same results at lower computational cost for very similar sequences.

### Verification of local alignment location on the human and chimpanzee genomes

Only reads having one unique best alignment each to the human and chimpanzee genomes are used in subsequent steps. The location of the human and chimp hits for each read is verified by using liftover [50] to map the coordinates of the human hit to the chimpanzee genome and the coordinate of the chimpanzee hit to the human genome. Only if the lifted coordinates overlap the respective alignment to at least 90% in both directions is the read used for divergence triangulation.

### Bitscore cutoff on second best hits

After the verification of local alignment locations, reads are filtered based on the bitscore difference between the best and second best hit to the human genome.

### Divergence estimates by triangulation

Divergence is calculated as the fraction of lineage-specific changes accumulated on the human lineage since the split from Neandertal to the changes accumulated on the chimpanzee lineage and that of the common ancestor of human and Neandertal before the split. We use the Neandertal sequence in a three-way Neandertal-human-chimpanzee alignment like an outgroup to count all changes that are only seen in human (Neandertal and chimpanzee being equal) and chimpanzee (human and Neandertal being equal). With the knowledge of the divergence time between human and chimpanzee and assuming no differences in substitution rate, the average divergence between human and Neandertals can be calculated as: Hs/(Hs + Cs) × D (Hs = human lineage specific changes, Cs = chimpanzee specific changes, D = 2 × average divergence between human and chimpanzee in million years (that is, 13 million years)).

## Abbreviations

bp: base pair; 3DP: three-dimensional dynamic programming.

## Authors' contributions

REG and KP designed the experiments. KP, REG and US wrote the code and performed the analyses. KP, US, MH, SP, JK and REG interpreted the results, discussed the implications and commented on the manuscripts at all stages. KP, JK and REG prepared the manuscript.

## Supplementary Material

Additional file 1 Figures S1 to S4 (Figure S4 uses data from [54]). Click here for file

Additional file 2 simulated datasets for 1 to 6 million years divergence from human in fasta format (also available from [47]). Each fasta record has a header that encodes the chromosome, start position and strand of the human location from which this fragment was sampled (that is, >N_chr15-75613688_-). Click here for file
