# Supplementary material for: Computational challenges in the analysis of ancient DNA
Source: Genome Biol. 2010 May 6;11(5):R47. doi: 10.1186/gb-2010-11-5-r47 (PMC2898072; doi:10.1186/gb-2010-11-5-r47)
Supplement: Additional file 1 — Figures S1 to S4 (Figure S4 uses data from [54]). [file gb-2010-11-5-r47-S1.pdf]

Challenges in the detection and analysis of ancient genomic DNA

Supplementary Figures

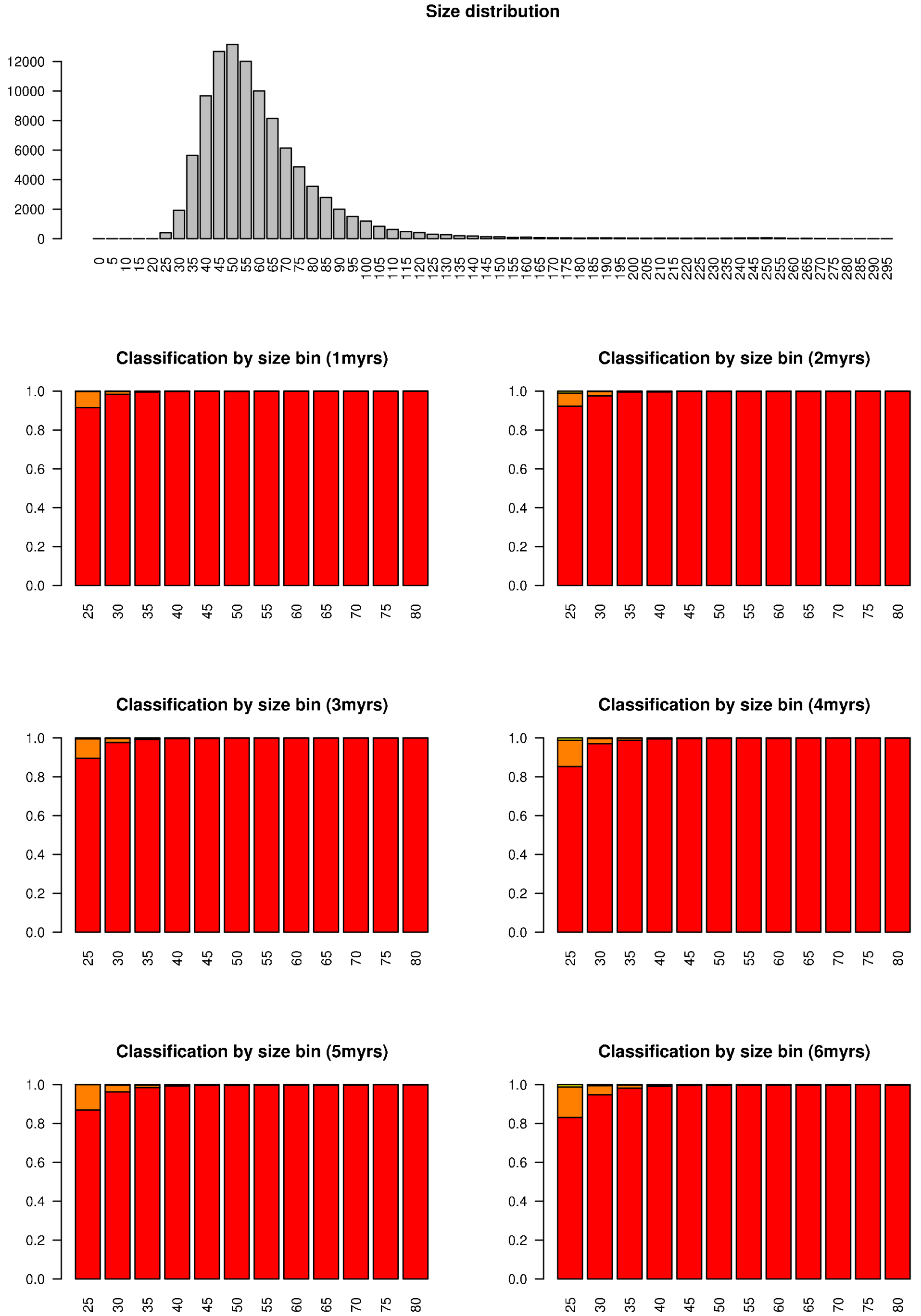

Figure 1: Size distribution and classification for simulated datasets. The top figure shows the size distribution of fragments for all simulated datasets. Below the size distribution, the number of fragments with best hit to human (red), no hit to any database (orange) and with a hit to any other species than Human (yellow) is shown for each simulation between one and six million years divergence.

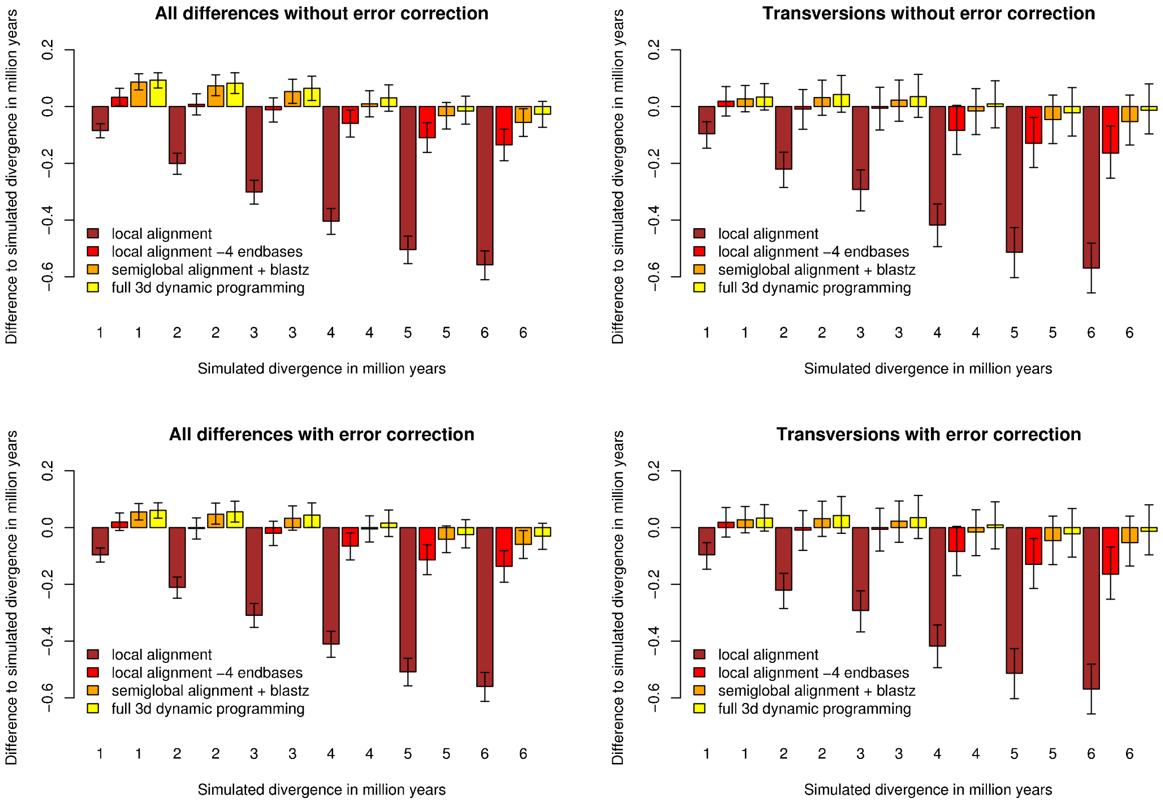

Figure 2: Deviation from simulated divergence for different alignment strategies. Left: divergence calculated using all substitutions, Right: divergence calculated using transversions only. Upper two figures show the raw divergence estimate, while the lower two figures use an error correction (see [9]).

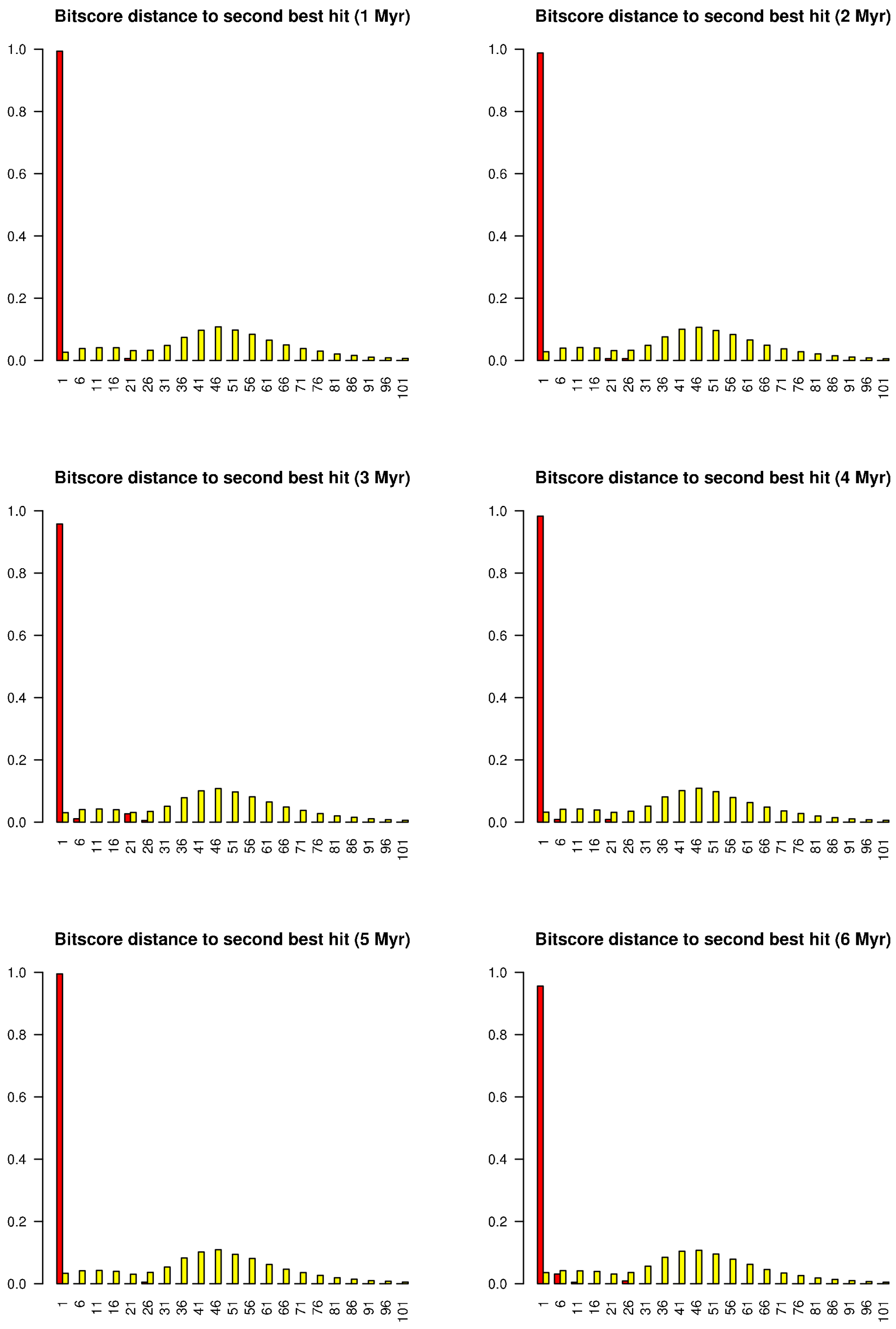

Figure 3: Histogram of differences in bitscore between best and second best local alignment hit for wrongly placed simulated reads (red) and all reads (yellow) for all simulated divergence times.

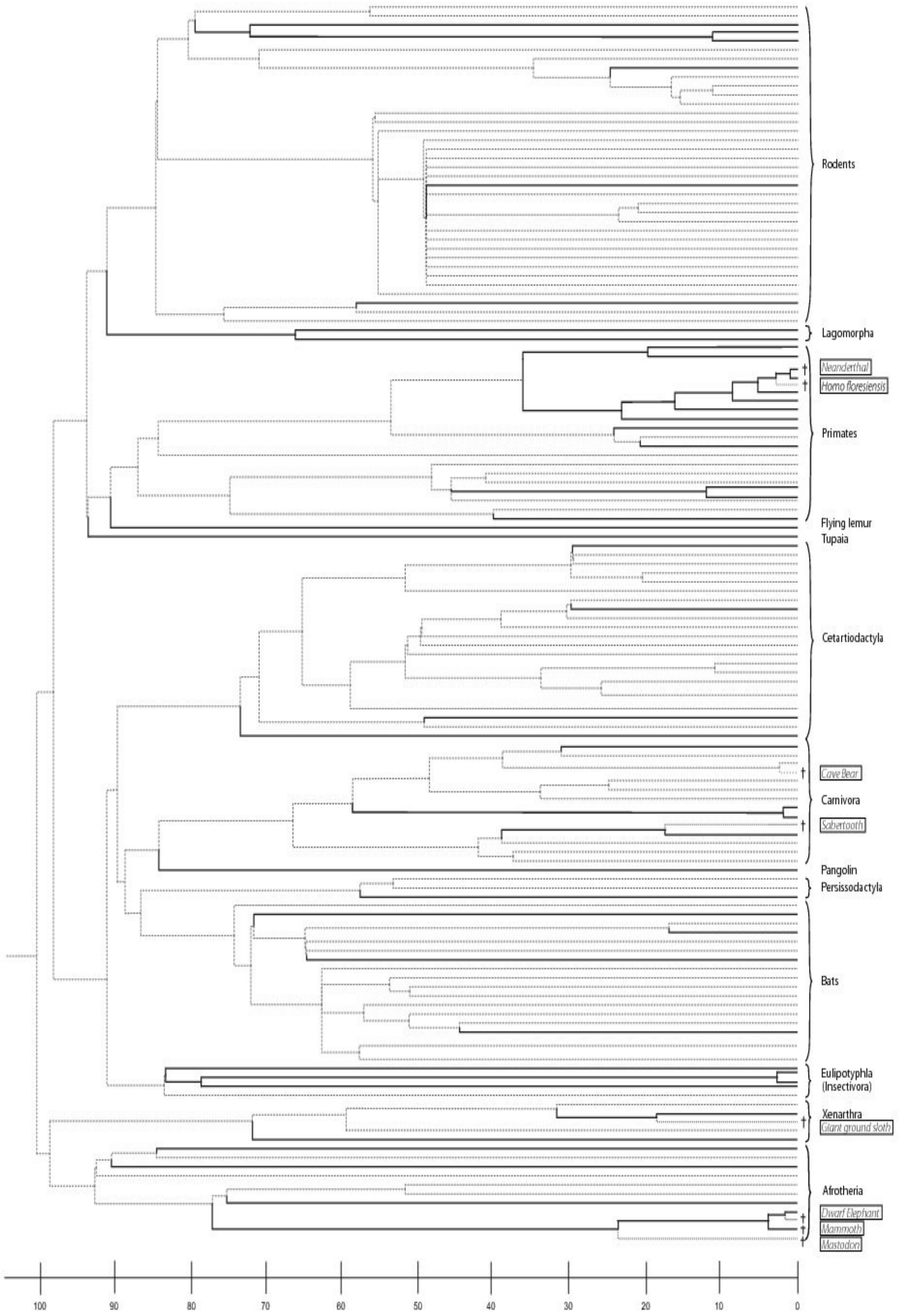

Figure 4: Family level tree for placental mammals (modified after [52] and other sources). Black continuous lines lead to families / species for which genome projects have been finished or are under way, dotted gray lines lead to families for which no genome sequencing projects are scheduled so far. Parenthesis summarize mammalian families, the names of the families are either given as Latin names, or, where available, as common names. Lineages ending with a cross lead to selected recently extinct species, with their names italicized and framed by boxes (see also table 1). The axis below the tree shows ages in million years.
